# A retrospective longitudinal study of 52 Finnish patients with X‐linked retinoschisis

**DOI:** 10.1111/aos.16776

**Published:** 2024-10-22

**Authors:** Mira A. Järvinen, Rigmor C. Baraas, Anna Majander, Michael P. Backlund, Julia Krootila, Maarjaliis Paavo, Päivi Lindahl, Kristiina Vasara, Eeva‐Marja Sankila, Tero T. Kivelä, Joni A. Turunen

**Affiliations:** ^1^ Department of Ophthalmology University of Helsinki and Helsinki University Hospital Helsinki Finland; ^2^ National Centre for Optics, Vision and Eye Care, Faculty of Health and Social Sciences University of South‐Eastern Norway Kongsberg Norway; ^3^ Eye Genetics Group Folkhälsan Research Center, Biomedicum Helsinki Helsinki Finland

**Keywords:** natural history, peripheral retinoschisis, Retinoschisin 1, RS1, X‐linked retinoschisis, XLRS

## Abstract

**Purpose:**

To describe clinical characteristics in Finnish patients with X‐linked retinoschisis (XLRS) longitudinally with emphasis on retinal morphology and genotype–phenotype correlations.

**Methods:**

A retrospective cohort study reviewed medical records from patients with genetically confirmed XLRS from the Department of Ophthalmology, Helsinki University Hospital. Best‐corrected visual acuity (BCVA), refraction, colour fundus photography, spectral‐domain optical coherence tomography and genetic information were collected.

**Results:**

Fifty‐two males were diagnosed at the median age of 7 years (range 1–57) and followed for a median of 8 years (range, 1–49). Baseline findings included macular retinoschisis in 92 (89%), macular atrophy in 25 (24%) and peripheral retinoschisis in 22 (21%) eyes. Vitreous haemorrhage occurred in 10 (10%) eyes, more frequently with peripheral schisis (*p* < 0.001). Nearly half of the patients, 22 (42%) were classified as visually impaired according to WHO. Median central retinal thickness was similar between initial (355 μm) and latest visits (360 μm) (*p* = 0.781). Low BCVA was associated with macular atrophy (*p* < 0.001), ellipsoid zone disruption (*p* = 0.007) and peripheral retinoschisis (*p* = 0.006). The three Finnish founder mutations c.214G >A, c.221G >T, and c.325G >C in exon 4 of retinoschisin 1 (*RS1*) were identified in 40 patients (77%). No associations were found between the genotypes and phenotypes.

**Conclusion:**

Three‐fourths of the patients carried the Finnish founder mutations in *RS1*, but we did not detect any genotype–phenotype association. Macular atrophy was associated with the poorest visual acuity. Ocular compilations were associated with peripheral retinoschisis, suggesting that these patients should be followed more frequently.

## 1 INTRODUCTION

X‐linked retinoschisis (XLRS; MIM **#**312700) is one of the most common types of early‐onset inherited retinal disease. It affects almost exclusively young men who develop a progressive decline in best‐corrected visual acuity (BCVA) from childhood, often detected during the first school years (Arden et al., 1988; George, Yates, Bradshaw, & Moore, 1995; George, Yates, & Moore, 1995; Molday et al., 2012; Rahman et al., 2020; Sieving et al., 1993). Global prevalence of XLRS is estimated to vary between 1 in 15 000 and 1 in 25 000 (Consortium, 1998; George, Yates, Bradshaw, & Moore, 1995; George, Yates, & Moore, 1995). Because of its population history, XLRS is more prevalent in Finland than elsewhere (Peltonen et al., 1999; Rudanko et al., 1993) with an estimated frequency exceeding 1:17000, translating to over 300 patients (Huopaniemi et al., 1999; Norio, 2003).

Bilateral schisis of the inner and outer retinal layers is the hallmark of XLRS. Radially arranged cystoid macular changes often create a spoke‐wheel pattern and dissociate the outer from the inner plexiform layer (Georgiou et al., 2022; Gerth et al., 2008). These changes can progress into macular atrophy over time. XLRS may also manifest with peripheral retinoschisis (Georgiou et al., 2022; Molday et al., 2012). Vitreous alterations may be present, such as vitreous veils and fibrous condensations extending from peripheral retinoschisis, as well as inner layer holes (George, Yates, Bradshaw, & Moore, 1995; George, Yates, & Moore, 1995; Molday et al., 2012). XLRS is commonly associated with hyperopia and astigmatism, and may coexist with strabismus or nystagmus (George, Yates, Bradshaw, & Moore, 1995; George, Yates, & Moore, 1995).

Qualitative abnormalities in the photoreceptor‐related layers, like shortened outer segment (OS) length and defects in cone outer segment tips (COST) line, occur frequently, can be detected by optical coherence tomography (OCT) and are associated with reduced BCVA (Apushkin et al., 2005; Bennett et al., 2016; Bowles et al., 2011; Cukras et al., 2018; Hahn et al., 2022; Ores et al., 2018; Yang et al., 2014). Disruption of the ellipsoid zone (EZ) is associated with decreased BCVA in children (Ling et al., 2020).

XLRS is caused by pathogenic variants in the retinoschisin 1 (*RS1*) gene (OMIM *300839), located on chromosome Xp22, that encodes a 224‐amino‐acid protein retinoschisin (Sauer et al., 1997). It is a disulphide‐linked, homo‐oligomeric extracellular protein that contains a discoidin domain crucial for retinal development and, presumably, for sustaining retinal cell‐to‐cell adhesion between photoreceptor and bipolar cells (Bush et al., 2016; Tantri et al., 2004; Weber et al., 2002). Defective retinoschisin generates retinoschisis, splitting of retinal layers (Lesch et al., 2008). The *RS1* gene has six exons. Over 190 pathogenic variants have been reported, typically missense in type (Huopaniemi et al., 1999; Molday et al., 2012). In Finland, over 95% of patients were found to carry one of three founder pathogenic variants in exon 4, c.214G >A, p.(Glu72Lys), c.325G >C, p.(Gly109Arg) or c.221G >T, p.(Gly74Val) (Huopaniemi et al., 1999).

Traditionally, BCVA was thought to decline during the first and second decades of life and then remain relatively stable until the fifth to sixth decade (Sieving et al., 1993). Recent large cohort studies reported, however, that after the initial decline BCVA continued to slowly decline with age (Georgiou et al., 2022; Hahn et al., 2022). The largest retrospective cohort study to date from the Netherlands and Belgium with 340 patients reported a decimal BCVA of 0.25–0.3 at the mean age of 29 years (Hahn et al., 2022). With increasing age, the visual impairment increases and the same study reported that at 60 years of age, severe visual impairment and blindness had a predicted prevalence of 35% and 25%, respectively (Hahn et al., 2022). Clear genotype–phenotype correlation has not been observed (Fortunato et al., 2023; Georgiou et al., 2022; Hahn et al., 2022). Instead, the clinical characteristics and progression of XLRS vary widely (Georgiou et al., 2022; Hahn et al., 2022). Complications of XLRS include vitreous haemorrhage (VH), retinal detachment (RD) and neovascular glaucoma (NVG) (Georgiou et al., 2022; Molday et al., 2012).

No effective medical treatment is available for XLRS. Carbonic anhydrase inhibitors, including oral acetazolamide and topical dorzolamide, may diminish macular cystoid changes, restore retinal anatomy and improve BCVA, but published results on that have been inconsistent (Fenner et al., 2023; Galantuomo et al., 2016; Genead et al., 2010; Pennesi et al., 2018; Verbakel et al., 2016; Walia et al., 2009). Although gene augmentation therapy has shown potential in preclinical studies using in vitro and rodent models, efforts to translate these successes into effective treatments for XLRS patients through clinical trials have not yet achieved desired outcomes but are being intensively developed (van der Veen et al., 2024).

For future development of treatments and clinical trials, it is crucial to understand the natural history of XLRS in detail. Here, we report the characteristics and long‐term clinical progression in a Finnish cohort with X‐linked retinoschisis (XLRS). The presence of three common founder pathogenic variants provided an opportunity to re‐investigate genotype–phenotype relationships.

## 2 MATERIALS AND METHODS

Eligible for this retrospective study were all Finnish patients diagnosed with, or reviewed for, XLRS at the Department of Ophthalmology, Helsinki University Hospital, Helsinki, Finland, between 1 January 2005 and 31 December 2018. Fifty patients with a pathogenic variant in the *RS1* gene and two patients with a typical and unambiguous clinical XLRS, X‐linked inheritance pattern, and previously detected pathogenic *RS1* variant in the family were enrolled.

The study was approved by the Helsinki University Hospital Institutional Review Board, and by the Regional Committee for Medical Research Ethics South East Norway and followed the tenets of the Declaration of Helsinki.

### 2.1 Patients and clinical data

Age, gender and the following variables were collected from patient charts: the age at diagnosis of XLRS and the last visit, the pathogenic variant and ocular complications during follow‐up. Ocular motility disorders, strabismus and nystagmus were charted. The type of retinoschisis was recorded as macular schisis, peripheral schisis and macular atrophy. BCVA on decimal scale was recorded from the first to the latest visit during follow‐up as available, at the maximum frequency of each 6 months. Decimal values were converted to logMAR. Finger counting was considered in decimal values as 0.01, hand motion 0.005 and light perception 0.002 according to published guidelines (Lange et al., 2009; Schulze‐Bonsel et al., 2006). Visual impairment adhered to WHO definitions (World Health Organization, Blindness and vision impairment, available at https://www.who.int/news‐room/fact‐sheets/detail/blindness‐and‐visual‐impairment). In short, mild visual impairment (6/12 >VA ≥6/18), moderate (6/18 >VA ≥6/60), severe (6/60 >VA ≥3/60) and blindness (VA, <3/60) in the better eye. The first available auto‐(AR) or manual (MR) refraction was the baseline refractive error, converted to the spherical equivalent (SER), and the latest one was also recorded.

### 2.2 Genetic data

When available, genetic data were primarily collected from patient records. When not available, the *RS1* (gene ID ENSG00000102104, transcript ENST00000379984.3, UniProt ID O15537) was sequenced for the three Finnish founder pathogenic variants, c.214G >A, p.(Glu72Lys), c.325G >C, p.(Gly109Arg) or c.221G >T, p.(Gly74Val), in HUSLAB, Helsinki University Hospital, Helsinki, Finland. If not detected, the whole *RS1* gene was sequenced by Asper Biogene A.S., Tartu, Estonia; Blueprint Genetics, Espoo, Finland; or Manchester Centre for Genomic Medicine, Manchester, United Kingdom.

### 2.3 Imaging

The digital colour fundus photographs were taken using Topcon CFP, TRC‐NW8F or TRC‐50DX fundus camera (Topcon), Clarus 500 (Zeiss) or Optos California (Optos PLC). The latest available image was used for the collection of clinical features. Spectral‐domain OCT (SD‐OCT) images were acquired with Spectralis OCT and OCT 2 (software Version 6.9a, Heidelberg Engineering) with standard settings (corneal radius 7.7 mm, axial eye length 22.5–26.5 mm). The central macular thickness (CMT) was recorded for the central 1 mm of the 9‐zone Early Treatment Diabetic Retinopathy Study (ETDRS) plot. Photoreceptor OS length, defined as the distance from the posterior surface of EZ, defined as the junction between photoreceptor inner and outer segments, to the anterior surface of the retinal pigment epithelium (RPE) on the latest visit was measured manually on the foveal horizontal image scan with the inbuilt manual calliper tool. If the EZ appeared disturbed, the extrapolated ghost line of its posterior border was traced as the anterior baseline for the OS length. EZ disruption, defined as an irregularity or a defect, and foveal atrophy, defined as the absence of EZ and interdigitation zone (IZ), were recorded from the latest scan and single baseline images.

### 2.4 Statistical analysis

Statistical analysis was performed using R‐software (version 3.4.3), combined with R Commander (R: A language and environment for statistical computing. R Foundation for Statistical Computing, Vienna, Austria. URL https://www.R‐project.org/). Continuous data were expressed as either means with standard deviations or medians with ranges. Inferential statistical methods such as correlation and comparisons between groups were used to assess associations between the variables. Groups were compared using the Mann–Whitney *U*‐test or Spearman correlation coefficient for continuous variables. Associations between the genotype and phenotype were assessed on multiple comparisons of Chi‐squared and Fisher's exact test. Five non‐founder pathogenic variants were categorized as a separate group for the analysis. Correlation between BCVA, refractive error and retinal morphology was assessed using an independent *t*‐test. The significance level was 0.05.

## 3 RESULTS

Fifty‐two male patients fulfilled the inclusion criteria. The median age of diagnosis, known for 50 patients, was 7 (range 1–57). The median length of follow‐up was 8 (range 1–49) years (Table 1).

**TABLE 1:** Clinical characteristics of 52 patients with X‐linked retinoschisis (XLRS).

Characteristics	Data
No of (male) patients/eyes (%)	52/104 (100)
Mean age onset/diagnosis (SD), years, *n* = 50	11 (13)
Median (range)	7 (1–57)
Mean follow‐up (SD), years, *n* = 52	11 (12)
Median (range)	8 (0–49)
Fundus findings, no of eyes (%)	
Macular schisis^a^	59/104 (57)
Peripheral schisis	0/104 (0)
Macular atrophy^b^	5/104 (5)
Macular schisis and macular atrophy	12/104 (12)
Macular schisis and peripheral schisis	14/104 (13)
Macular atrophy and peripheral schisis	1/104 (1)
Macular schisis, −atrophy and peripheral schisis	7/104 (7)
Normal fundus	6/104 (6)
Strabismus, no of patients (%)	10/52 (19)
Nystagmus, no of patients (%)	3/52 (6)
Spherical equivalent refractive error (D) no of eyes, last visit (*n* = 53)	
Mean (SD)	1.99 (2.11)
Median (range)	1.13 (−0.88–7.13)

Abbreviations: AR, Autorefraction; SD, Standard deviation; SR, Subjective refraction.

^a^ Foveal schisis, parafoveal schisis or combined.

^b^ Absent ellipsoid zone (EZ) and interdigitation zone (IZ).

**FIGURE 1:**
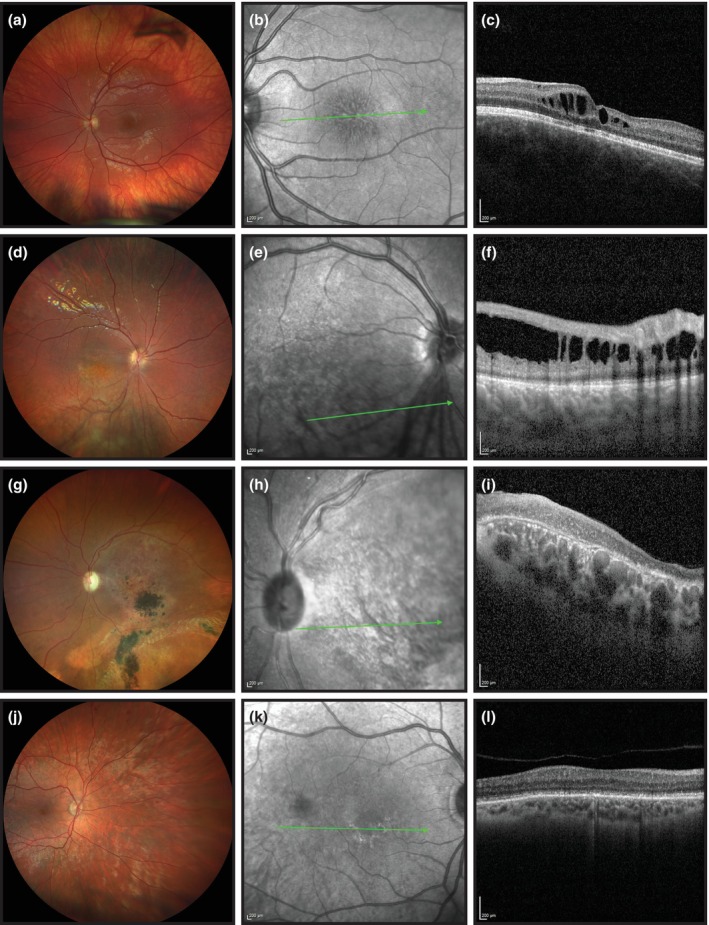
Typical findings in patients with X‐linked retinoschisis (XLRS). (a) The fundus photography and (b) infrared fundus photography of the left eye of an 11‐year‐old boy show a macular spoke‐wheel pattern. The vitreous veil can be seen in the superior vitreous. (b) Infrared fundus photography. (c) Spectral‐domain optical coherence tomography (OCT) scan shows splitting and disarrangement of retinal layers and ellipsoid zone disruption. Foveal and parafoveal retinoschisis are located mainly in the inner nuclear layer and outer plexiform layer. (d) The fundus photography and (e) infrared fundus photography of the right eye of a 15‐year‐old boy showing macular pigmentary changes and inferior peripheral retinoschisis. (f) OCT image shows inferior macular retinoschisis. (g) The fundus photography and (h) infrared fundus photography of the right eye of a 44‐year‐old man show macular atrophy and pigmentary deposits. (i) OCT shows macular atrophy with the absence of the ellipsoid zone and the interdigitation zone. (j) The fundus photography and (k) infrared fundus photography of the left eye of a 67‐year‐old man show modest pigmentary alterations in the macula. The retina shows a typical golden sheen or tapetal reflex of the fundus outside the macula. (l) OCT image shows mild atrophy and disruptions in the ellipsoid zone and typical macular schisis and cysts have disappeared.

At baseline, macular retinoschisis was recorded in 59 (57%) eyes and in the latest fundus image in 22 (78%) of 28 eyes, respectively (Figure 1a–c). Macular atrophy (Figure 1j–l) was observed in 25 (24%) eyes, and peripheral retinoschisis in 22 (21%) eyes. Macular atrophy was associated with older age (*p* < 0.001, Mann–Whitney *U*‐test). The distribution of different types of retinal characteristics varied. Macular retinoschisis was the only finding in 57%, and macular atrophy in 5% of the eyes. Strabismus was diagnosed in 10 (19%) patients during follow‐up, six with esotropia and four with exotropia. Three (6%) patients had nystagmus. Association analysis of different phenotypes is presented in Table 2 and the distribution of retinal characteristics in Table S1.

**TABLE 2:** The association analysis of the genotypes and phenotypes in the patients with X‐linked retinoschisis.

Phenotype	Value	*p*‐value
Macular schisis	2.87	0.76
Peripheral schisis	2.68	0.81
Macular atrophy	2.84	0.77
EZ disruption	4.55	0.52
Strabismus	8.29	0.25
Nystagmus	1.82	>0.99
Vitreous haemorrhage	1.25	0.94
Visual impairment	5.14	0.28

*Note*: Chi‐squared test, multiple comparisons, Fisher's exact test.

Abbreviation: EZ, ellipsoid zone.

### 3.1 Visual function

**FIGURE 2:**
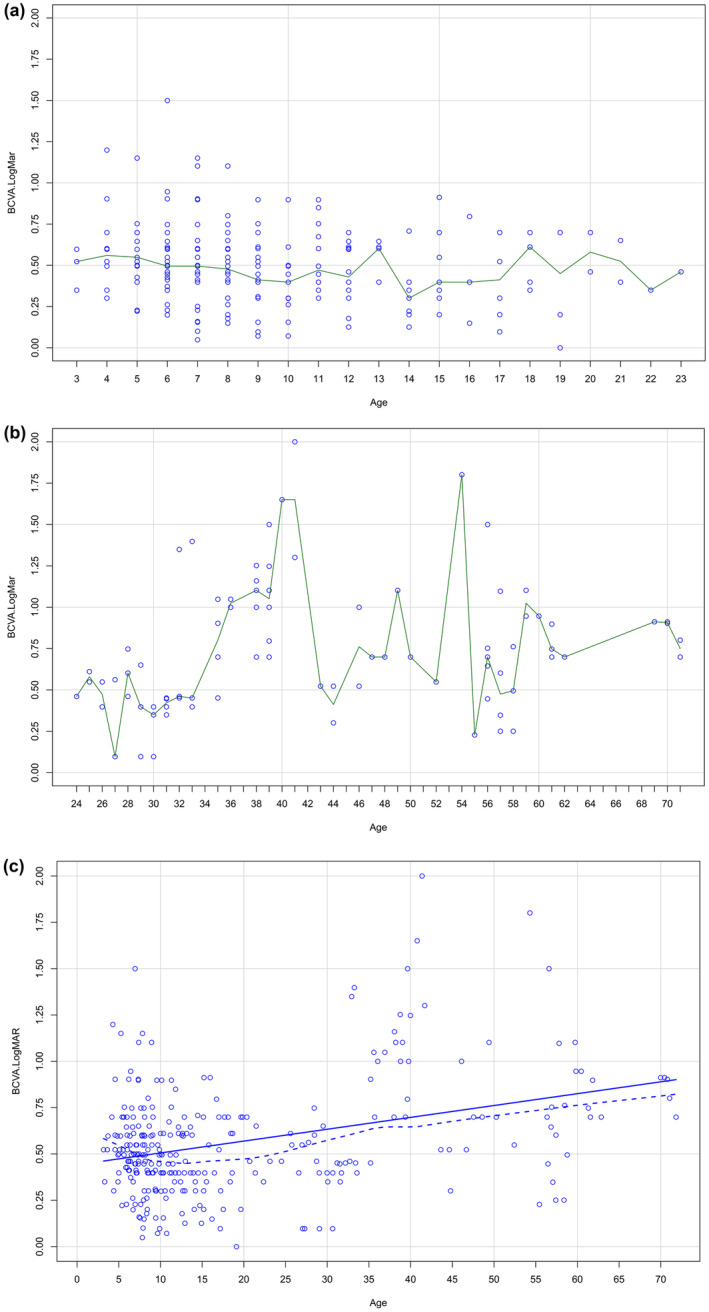
(a). The scatter plot of median best‐corrected visual acuity (BCVA, right eye) by the age of 52 Finnish patients with X‐linked retinoschisis (XLRS) before 24 years of age. No difference in BCVA from infancy to adulthood. (b). The scatter plot of the patients 24 years or older. (c). The scatter plot of median BCVA (right eye) for all ages. The solid line presents linear regression of best‐corrected visual acuity (BCVA) and age and the dashed line presents a loss curve.

Visual function had been monitored for a median of 8 years (range 0.5–49). At the time of the latest visit, 15 (28%) of 52 patients were older than 30 years of age. Baseline BCVA was 0.58 logMAR (SD 0.40, *n* = 50, median 0.50) in RE and the latest mean BCVA was 0.59 logMAR (SD 0.46, *n* = 50, median 0.50) in RE. Initial and latest visit BCVA were highly variable between patients. BCVA ranged from light perception (=2.7 logMAR) to 0 logMAR. The mean BCVA on the latest visit was 0.57 (SD 0.47, *n* = 44) LogMAR in RE for patients with macular schisis, 0.91 (SD 0.65, *n* = 12) for patients with macular atrophy, and 0.98 (SD 0.80, *n* = 10) for patients with peripheral schisis. The age‐related median BCVA remained relatively stable before 24 years of age (Figure 2a). In the patients under 20 years, we did not observe correlation between BCVA and age (R, 0.229, *p* = 0.063, Spearman's correlation coefficient) in RE. After 20 years the BCVA declined and was correlated with age (R, 0.554, *p* = 0.030, Spearman's correlation coefficient) in RE. An association between BCVA and macular atrophy (*p* = 0.006, Mann–Whitney *U‐*test) in RE was found. We did not find association between BCVA and macular schisis (*p* = 0.36), peripheral retinoschisis (*p* = 0.07) or EZ disruption (*p* = 0.17). At the last visit, nearly half of the patients, 22 (42%) were classified as visually impaired according to WHO. Patients with acute VH were excluded from these data.

At the initial visit, SER was measured to be hypermetropic in 80% (69 eyes) with AR and in 60% (51 eyes) with MR. Emmetropia and myopia increased in frequency towards the latest visit (Table S2). As a whole, 96% of eyes measured with AR (51 eyes) and 82% with MR (52 eyes) remained emmetropic or hyperopic until the end of the follow‐up, whereas myopic eyes accounted only for 4% of those measured with AR (two eyes) versus 17% with MR (11 eyes).

### 3.2 Optical coherence tomography

Baseline SD‐OCT scans were available for 44 patients from at least one eye (RE was available at the baseline for 41 and last visit for 31, respectively, and only RE measures are reported below). The time between the first and the latest SD‐OCT ranged from 0.5 to 8.5 years (mean 3.2, *n* = 31). Median CMT did not differ at the initial and latest visit 355 (range 172–875) μm, versus 360 (range 160–637) μm; (*p =* 0.781, paired *t*‐test). The initial median CMT in patients with macular retinoschisis was 365 (range 172–875, *n* = 35) μm, and last visit 384 (range 160–637, *n* = 29) μm. In patients with atrophy, initial median CMT was 204 (range 167–256, *n* = 9) μm and the last visit 200 (range 160–349, *n* = 6) μm. CMT appeared thinner in patients with atrophy (OD, *p* < 0.001, independent *t*‐test). The median OS length on the latest visit (*n* = 28) was 25.79 (range 11–38) μm. EZ disruption was observed in 24 eyes and foveal atrophy in 12 eyes.

The AR was not correlated with CMT or OS length (Table S3). The same was true of SR and CMT or OS, and of EZ disruption.

### 3.3 Genetic characteristics

**FIGURE 3:**
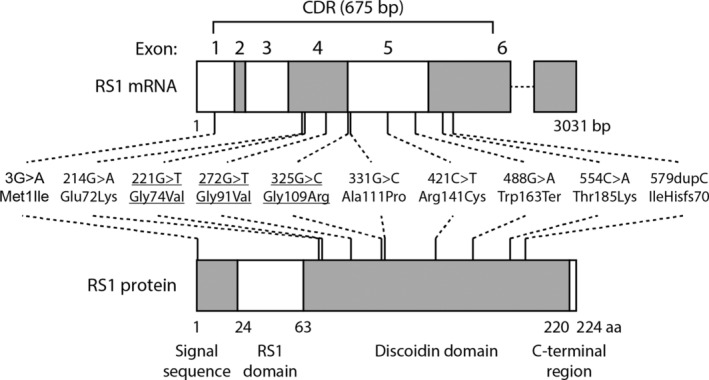
A schematic representation of the identified variants in the *RS1* gene in Finnish patients. The locations of the variants (middle panel) in *RS1* mRNA transcript (top panel) and RS1 protein (bottom panel) are indicated. Different exons and protein motifs are shown as altering white and grey boxes. The width of the box is relative to the length of the exon or protein domain, except for exon 6, which is shortened after the coding region (CDR) by a dashed line. The 675 bp CDR between bases 41–715 of the 3031 bp mRNA transcript is indicated above the figure. The names of the RS1 protein domains are shown below the figure. The Finnish founder mutations are underlined.

The most frequent pathogenic variants in *RS1* were: c.214G >A, p.(Glu72Lys), 24 (46%) patients; c.221G >T, p.(Gly74Val), 11 (21%) patients; and c. 325G >C, p.(Gly109Arg), five (10%) patients. Overall, 40 (77%; 95% confidence interval [CI] 66–88) of tested patients had one of these known Finnish founder variants (Table S4) (Huopaniemi et al., 1999). Three (6%) patients had a previously reported pathogenic variant in exon 6: c.554C >A p.(Thr185Lys) (Huopaniemi et al., 1999). Additionally detected pathogenic variants were c.3G >A, p.(Met1Ile); c.272G >T, p.(Gly91Val); c.331G >C, p.(Ala111Pro); c.421C >T, p.(Arg141Cys); c.488G >A, p.(Trp163Ter); c.488G >A p. (Trp163*); and c.579dupC, p.(IleHisfs70). The variants c.3G >A (Xiao et al., 2016), c.421C >T (Consortium, 1998), c.488G >A, (Wang et al., 2015), and c.579dupC (Neriyanuri et al., 2016) were reported earlier. To the best of our knowledge, the variants c.272G >T and c.331G >C are not described previously. The location of the variants in relation to the domains in the RS1 protein is presented in Figure 3. No associations were detected between genotypes and phenotypes (Table 2).

### 3.4 Complications

Vitreous haemorrhages were recorded in 10 (10%) of the eyes and eight (15%) of patients, none had a RD or NVG. The median age at first VH (*n* = 7) was 10 years (range, 0.5–24). Most complications were unilateral, however, two patients had VH in both eyes during follow‐up. VH was recurrent in three of the 10 eyes, occurring twice in two eyes and thrice in one eye. VH was more frequent in patients with peripheral retinoschisis (7/104 (7%), *p <* 0.001, Fisher's exact test) compared to patients with macular retinoschisis. Of 22 eyes with peripheral schisis, seven (32%) had VH at least once. Macular schisis (2/71 (3%), *p =* 0.35), macular atrophy (1/17 (5%), *p =* 0.51) or EZ‐disruption (4/50 (8%), *p =* 0.652*)* were not associated with VH. Five patients with VH carried the genotype c.214G > A, two c.221G > T and one c.331G > C.

### 3.5 Carbonic anhydrase inhibitor treatment

Seven (13%) patients were treated for 2 weeks to 1 year 4 months with oral (*n* = 6) or topical (*n* = 1) carbonic anhydrase inhibitors during follow‐up. BCVA varied throughout the treatment period (Table S5). One patient gained two logMAR lines in his left eye within a 2‐week period (from 0.4 to 0.2 on the logMAR scale). The maximum decline in BCVA was from 0.32 to 0.5 logMAR during treatment. The overall change in BCVA was −0.05 log unit in all treated eyes.

## 4 DISCUSSION

This is the first longitudinal report of structural and functional characteristics of a cohort comprising about one‐sixth of the estimated number of patients with XLRS in Finland. BCVA varied markedly between individuals, with an initial decline in childhood and a continued decline during the third decade of life. Peripheral retinoschisis was associated with vitreous haemorrhage.

Vision decline, as measured by visual acuity, is one of the first signs of XLRS. There has been some uncertainty as to if BCVA will remain stable after the initial decline during first and second decades of life (Eksandh et al., 2000; Wood et al., 2019). Here, we confirm that BCVA continues to decline during the third decade of life, supporting the results from a large European cohort study (Hahn et al., 2022). It has been also reported earlier that BCVA did not correlate with age in XLRS (Lesch et al., 2008). In our study, the BCVA and age were not correlated in the patients under 20 years old, but in the patients over 20 years BVCA declined. Mean BCVA was close to the WHO definition of low vision 0.63 logMAR (SD 0.42), similar to two recent reports (0.65 and 0.6 logMAR) (Georgiou et al., 2022; Hahn et al., 2022). Almost half of our patients (47%) were visually impaired according to WHO. The predicted prevalence of severe visual impairment and blindness by the age of 60 was 35% and 25%, respectively, which was similar to the European cohort (Hahn et al., 2022). The high prevalence in our study might be related to the fact that patients with less severe vision loss are not referred to and followed in specialized medical care, causing an overrepresentation of visual impairment. The association between low BCVA and macular atrophy, EZ disruption, and peripheral retinoschisis are likely due to that peripheral retinoschisis typically occurs in combination with macular schisis or macular atrophy, and with a higher risk of complications affecting visual acuity, especially VH.

We found no correlation between OS length and BCVA or between CMT and BCVA as has been earlier reported (Apushkin et al., 2005; Bennett et al., 2016; Cukras et al., 2018; Hahn et al., 2022; Ores et al., 2018; Yang et al., 2014). The mean OS length was shorter (26.1 (SD 7.0) μm, *p* = <0.0001) than recently reported (36.4 (SD 6.9) μm) (Georgiou et al., 2022), but longer than in two other reports (21.2 (SD 8.3) μm) and (24.2 (SD 9.2) μm) (Hahn et al., 2022; Ores et al., 2018). Both interdigitation zone (IZ) quality and OS length have been suggested to be more representative considering visual function than CMT, because CMT is affected by the presence of macular cysts. Qualitative defects of IZ and EZ are a typical clinical manifestation in XLRS (Yang et al., 2014). It is noteworthy that in our study only 6% of eyes had EZ disruptions only, with the absence of all the other retinal XLRS‐characteristics at the last visit. These patients had macular retinoschisis reported at the earlier stage of the disease.

A large variety of clinical severity of the disease was evident. The most frequent retinal change was intraretinal macular cysts. Radially arranged spoke‐wheel folds were observed in colour fundus images in 78% of eyes, and cystic macular changes were observed in 88% of eyes. Macular schisis has been described to develop into macular atrophy with age (George, Yates, Bradshaw, & Moore, 1995; George, Yates, & Moore, 1995) and this was observed in 23% of eyes, together with declining BCVA, corroborating previous reports (Lesch et al., 2008). Peripheral retinoschisis was observed in 19% of eyes, again like in previous reports (Eksandh et al., 2000; Ores et al., 2018).

We detected nine different pathogenic variants in the *RS1* gene, the most common being expectedly the three founder pathogenic variants (Huopaniemi et al., 1999) that accounted for 82% of tested patients, slightly less the previously detected of 95% (Huopaniemi et al., 1999). Two novel variants, c.272G >T and c.331G >C, were detected. Awareness of genetic aetiology is important, particularly for asymptomatic female carriers. The phenotype could not be predicted from the genotype like recent studies have reported (Fortunato et al., 2023; Georgiou et al., 2022; Hahn et al., 2022).

Ocular complications increase the risk of visual impairment (Sen et al., 2018; Wood et al., 2019). RD did not develop in our patients during our follow‐up. VH was found in 15% of patients, falling within the range of previous estimates of 3%–40% (Molday et al., 2012; Tantri et al., 2004). Of all patients with peripheral retinoschisis, 32% presented with VH, in line with recent studies (Fahim et al., 2017; Georgiou et al., 2022; Zhao et al., 2018). Therefore, patients manifesting with peripheral retinoschisis might need more detailed education of symptoms of VH (and RD), and possibly more frequent follow‐up than those without peripheral retinoschisis.

Refraction remained stable during follow‐up, and as expected, confirmed current consensus that patients with XLRS mostly have hyperopic refraction. Frequency of myopia increased towards the last visit but remained low.

Our study has limitations, especially its retrospective nature. The extent and accuracy of data in patient charts variy. Variable follow‐up times and various types of vision charts used affect the reliability of longitudinal BCVA data. OCT characteristics were analysed without having axial length and keratometry values of each patient. Knowledge of the axial length could have increased the reliability of the CMT estimates. Collecting data over the central 1 degree instead of 1 mm could have avoided issues with lateral scaling and increased accuracy (Odell et al., 2011), but it was not possible to implement with the available software version. These factors may increase the variability of the size of the actual measured area or induce bias on automatically measured CMT. Secondly, OCT images were taken with different scan patterns and the precise foveal position might have been missed and caused bias in estimated OS length and CMT. Despite these restrictions, our results confirm previous research, where applicable, which supports the importance of assessing such a dataset and presents a realistic comprehensive overview of the characteristics of XLRS in Finland. Our study also provides insights for further research such as more detailed prospective analysis of photoreceptor layer microstructure and its relationship to visual function, implementing a standardized OCT scan protocol and more detailed OCT analysis.

In conclusion, the genetic variant does not primarily seem to influence the disease phenotype, and thus other still unknown factors, genetic and/or environmental, modify the clinical presentation. The peripheral schisis was associated with VH, and the patients with peripheral schisis should be counselled accordingly. Visual acuity continued to decline already in the third decade of life. Almost half of the patients were visually impaired according to WHO, making XLRS a real burden for the patients, underlining the importance of continued research to find viable treatment options for them.

## FUNDING INFORMATION

The Folkhälsan Research Foundation Helsinki, Finland; the Eye and Tissue; Bank Foundation, Helsinki, Finland; and the Eye Foundation, Helsinki, Finland.

## CONFLICT OF INTEREST STATEMENT

Joni A. Turunen received lecture fees from Thea Finland, Santen. Finland, and has served on the advisory board of Novartis Finland. Tero T. Kivelä received lecture fees from Santen Finland, unrelated to the present work. The following authors report no financial conflict of interest: Mira A. Järvinen, Rigmor C. Baraas, Anna Majander, Michael P. Backlund, Julia Krootila, Maarjaliis Paavo, Päivi Lindahl, Kristiina Vasara, Eeva‐Marja Sankila.

## Supporting information


Table S1.

Table S2.

Table S3.

Table S4.

Table S5.

## References

[aos16776-bib-0001] Apushkin, M.A. , Fishman, G.A. & Janowicz, M.J. (2005) Correlation of optical coherence tomography findings with visual acuity and macular lesions in patients with X‐linked retinoschisis. Ophthalmology, 112, 495–501. 15745780 10.1016/j.ophtha.2004.08.027

[aos16776-bib-0002] Arden, G.B. , Gorin, M.B. , Polkinghorne, P.J. , Jay, M. & Bird, A.C. (1988) Detection of the carrier state of X‐linked retinoschisis. American Journal of Ophthalmology, 105, 590–595. 3377039 10.1016/0002-9394(88)90049-9

[aos16776-bib-0003] Bennett, L.D. , Wang, Y.Z. , Klein, M. , Pennesi, M.E. , Jayasundera, T. & Birch, D.G. (2016) Structure/psychophysical relationships in X‐linked retinoschisis. Investigative Ophthalmology & Visual Science, 57, 332–337. 26830370 10.1167/iovs.15-18354 PMC4736741

[aos16776-bib-0004] Bowles, K. , Cukras, C. , Turriff, A. , Sergeev, Y. , Vitale, S. , Bush, R.A. et al. (2011) X‐linked retinoschisis: RS1 mutation severity and age affect the ERG phenotype in a cohort of 68 affected male subjects. Investigative Ophthalmology & Visual Science, 52, 9250–9256. 22039241 10.1167/iovs.11-8115 PMC3302432

[aos16776-bib-0005] Bush, M. , Setiaputra, D. , Yip, C.K. & Molday, R.S. (2016) Cog‐wheel octameric structure of RS1, the Discoidin domain containing retinal protein associated with X‐linked retinoschisis. PLoS One, 11, e0147653. 26812435 10.1371/journal.pone.0147653 PMC4728063

[aos16776-bib-0006] Consortium TR . (1998) Functional implications of the spectrum of mutations found in 234 cases with X‐linked juvenile retinoschisis. Human Molecular Genetics, 7, 1185–1192. 9618178 10.1093/hmg/7.7.1185

[aos16776-bib-0007] Cukras, C.A. , Huryn, L.A. , Jeffrey, B.G. , Turriff, A. & Sieving, P.A. (2018) Analysis of anatomic and functional measures in X‐linked retinoschisis. Investigative Ophthalmology & Visual Science, 59, 2841–2847. 30025115 10.1167/iovs.17-23297 PMC5987578

[aos16776-bib-0008] Eksandh, L.C. , Ponjavic, V. , Ayyagari, R. , Bingham, E.L. , Hiriyanna, K.T. , Andreasson, S. et al. (2000) Phenotypic expression of juvenile X‐linked retinoschisis in Swedish families with different mutations in the XLRS1 gene. Archives of Ophthalmology, 118, 1098–1104. 10922205 10.1001/archopht.118.8.1098

[aos16776-bib-0009] Fahim, A.T. , Ali, N. , Blachley, T. & Michaelides, M. (2017) Peripheral fundus findings in X‐linked retinoschisis. The British Journal of Ophthalmology, 101, 1555–1559. 28348004 10.1136/bjophthalmol-2016-310110

[aos16776-bib-0010] Fenner, B.J. , Russell, J.F. , Drack, A.V. , Dumitrescu, A.V. , Sohn, E.H. , Russell, S.R. et al. (2023) Long‐term functional and structural outcomes in X‐linked retinoschisis: implications for clinical trials. Front Med (Lausanne), 10, 1204095. 37396901 10.3389/fmed.2023.1204095 PMC10310546

[aos16776-bib-0011] Fortunato, P. , Pagliazzi, A. , Bargiacchi, S. , Marziali, E. , Sodi, A. , Caputo, R. et al. (2023) X‐linked retinoschisis: mutation spectrum and genotype‐phenotype relationship in an Italian pediatric cohort. Ophthalmic Genetics, 44, 35–42. 36377647 10.1080/13816810.2022.2141790

[aos16776-bib-0012] Galantuomo, M.S. , Fossarello, M. , Cuccu, A. , Farci, R. , Preising, M.N. , Lorenz, B. et al. (2016) Rebound macular edema following oral acetazolamide therapy for juvenile X‐linked retinoschisis in an Italian family. Clinical Ophthalmology, 10, 2377–2382. 27932860 10.2147/OPTH.S114568 PMC5135400

[aos16776-bib-0013] Genead, M.A. , Fishman, G.A. & Walia, S. (2010) Efficacy of sustained topical dorzolamide therapy for cystic macular lesions in patients with X‐linked retinoschisis. Archives of Ophthalmology, 128, 190–197. 20142541 10.1001/archophthalmol.2009.398

[aos16776-bib-0014] George, N.D. , Yates, J.R. , Bradshaw, K. & Moore, A.T. (1995) Infantile presentation of X linked retinoschisis. The British Journal of Ophthalmology, 79, 653–657. 7662629 10.1136/bjo.79.7.653 PMC505192

[aos16776-bib-0015] George, N.D. , Yates, J.R. & Moore, A.T. (1995) X linked retinoschisis. The British Journal of Ophthalmology, 79, 697–702. 7662639 10.1136/bjo.79.7.697 PMC505202

[aos16776-bib-0016] Georgiou, M. , Finocchio, L. , Fujinami, K. , Fujinami‐Yokokawa, Y. , Virgili, G. , Mahroo, O.A. et al. (2022) X‐linked retinoschisis: deep phenotyping and genetic characterization. Ophthalmology, 129, 542–551. 34822951 10.1016/j.ophtha.2021.11.019

[aos16776-bib-0017] Gerth, C. , Zawadzki, R.J. , Werner, J.S. & Heon, E. (2008) Retinal morphological changes of patients with X‐linked retinoschisis evaluated by Fourier‐domain optical coherence tomography. Archives of Ophthalmology, 126, 807–811. 18541843 10.1001/archopht.126.6.807 PMC2612690

[aos16776-bib-0018] Hahn, L.C. , van Schooneveld, M.J. , Wesseling, N.L. , Florijn, R.J. , Ten Brink, J.B. , Lissenberg‐Witte, B.I. et al. (2022) X‐linked retinoschisis: novel clinical observations and genetic Spectrum in 340 patients. Ophthalmology, 129, 191–202. 34624300 10.1016/j.ophtha.2021.09.021

[aos16776-bib-0019] Huopaniemi, L. , Rantala, A. , Forsius, H. , Somer, M. , de la Chapelle, A. & Alitalo, T. (1999) Three widespread founder mutations contribute to high incidence of X‐linked juvenile retinoschisis in Finland. European Journal of Human Genetics, 7, 368–376. 10234514 10.1038/sj.ejhg.5200300

[aos16776-bib-0020] Lange, C. , Feltgen, N. , Junker, B. , Schulze‐Bonsel, K. & Bach, M. (2009) Resolving the clinical acuity categories “hand motion” and “counting fingers” using the Freiburg visual acuity test (FrACT). Graefe's Archive for Clinical and Experimental Ophthalmology, 247, 137–142. 10.1007/s00417-008-0926-0 18766368

[aos16776-bib-0021] Lesch, B. , Szabo, V. , Kanya, M. , Somfai, G.M. , Vamos, R. , Varsanyi, B. et al. (2008) Clinical and genetic findings in Hungarian patients with X‐linked juvenile retinoschisis. Molecular Vision, 14, 2321–2332. 19093009 PMC2603250

[aos16776-bib-0022] Ling, K.P. , Mangalesh, S. , Tran‐Viet, D. , Gunther, R. , Toth, C.A. & Vajzovic, L. (2020) Handheld spectral domain optical coherence tomography findings of X‐linked retinoschisis in early childhood. Retina, 40, 1996–2003. 31764609 10.1097/IAE.0000000000002688 PMC8896576

[aos16776-bib-0023] Molday, R.S. , Kellner, U. & Weber, B.H. (2012) X‐linked juvenile retinoschisis: clinical diagnosis, genetic analysis, and molecular mechanisms. Progress in Retinal and Eye Research, 31, 195–212. 22245536 10.1016/j.preteyeres.2011.12.002 PMC3334421

[aos16776-bib-0024] Neriyanuri, S. , Dhandayuthapani, S. , Arunachalam, J.P. & Raman, R. (2016) Phenotypic characterization of X‐linked retinoschisis: clinical, electroretinography, and optical coherence tomography variables. Indian Journal of Ophthalmology, 64, 513–517. 27609164 10.4103/0301-4738.190140 PMC5026077

[aos16776-bib-0025] Norio, R. (2003) Finnish disease heritage II: population prehistory and genetic roots of Finns. Human Genetics, 112, 457–469. 12627296 10.1007/s00439-002-0876-2

[aos16776-bib-0026] Odell, D. , Dubis, A.M. , Lever, J.F. , Stepien, K.E. & Carroll, J. (2011) Assessing errors inherent in OCT‐derived macular thickness maps. Journal of Ophthalmology, 2011, 692574. 21869920 10.1155/2011/692574 PMC3157761

[aos16776-bib-0027] Ores, R. , Mohand‐Said, S. , Dhaenens, C.M. , Antonio, A. , Zeitz, C. , Augstburger, E. et al. (2018) Phenotypic characteristics of a French cohort of patients with X‐linked retinoschisis. Ophthalmology, 125, 1587–1596. 29739629 10.1016/j.ophtha.2018.03.057

[aos16776-bib-0028] Peltonen, L. , Jalanko, A. & Varilo, T. (1999) Molecular genetics of the Finnish disease heritage. Human Molecular Genetics, 8, 1913–1923. 10469845 10.1093/hmg/8.10.1913

[aos16776-bib-0029] Pennesi, M.E. , Birch, D.G. , Jayasundera, K.T. , Parker, M. , Tan, O. , Gurses‐Ozden, R. et al. (2018) Prospective evaluation of patients with X‐linked retinoschisis during 18 months. Investigative Ophthalmology & Visual Science, 59, 5941–5956. 30551202 10.1167/iovs.18-24565 PMC6295939

[aos16776-bib-0030] Rahman, N. , Georgiou, M. , Khan, K.N. & Michaelides, M. (2020) Macular dystrophies: clinical and imaging features, molecular genetics and therapeutic options. The British Journal of Ophthalmology, 104, 451–460. 31704701 10.1136/bjophthalmol-2019-315086 PMC7147237

[aos16776-bib-0031] Rudanko, S.L. , Flage, T. , Hansen, E. , Rosenberg, T. , Viggosson, G. & Riise, R. (1993) Visual impairment in Nordic children. V. X‐linked juvenile retinoschisis. Acta Ophthalmologica, 71, 586–589. 8109204 10.1111/j.1755-3768.1993.tb04646.x

[aos16776-bib-0032] Sauer, C.G. , Gehrig, A. , Warneke‐Wittstock, R. , Marquardt, A. , Ewing, C.C. , Gibson, A. et al. (1997) Positional cloning of the gene associated with X‐linked juvenile retinoschisis. Nature Genetics, 17, 164–170. 9326935 10.1038/ng1097-164

[aos16776-bib-0033] Schulze‐Bonsel, K. , Feltgen, N. , Burau, H. , Hansen, L. & Bach, M. (2006) Visual acuities “hand motion” and “counting fingers” can be quantified with the freiburg visual acuity test. Investigative Ophthalmology & Visual Science, 47, 1236–1240. 16505064 10.1167/iovs.05-0981

[aos16776-bib-0034] Sen, P. , Agarwal, A. , Bhende, P. , Gopal, L. , Bhende, M. , Rishi, P. et al. (2018) Outcome of vitreoretinal surgery for rhegmatogenous retinal detachment in X‐linked juvenile retinoschisis. Indian Journal of Ophthalmology, 66, 1825–1831. 30451188 10.4103/ijo.IJO_607_18 PMC6256875

[aos16776-bib-0035] Sieving, P.A. , MacDonald, I.M. & Hoang, S. (1993) X‐linked congenital retinoschisis. In: Adam, M.P. , Feldman, J. , Mirzaa, G.M. , Pagon, R.A. , Wallace, S.E. , Bean, L.J.H. et al. (Eds.) GeneReviews(R). Seattle (WA): University of Washington. 20301401

[aos16776-bib-0036] Tantri, A. , Vrabec, T.R. , Cu‐Unjieng, A. , Frost, A. , Annesley, W.H., Jr. & Donoso, L.A. (2004) X‐linked retinoschisis: a clinical and molecular genetic review. Survey of Ophthalmology, 49, 214–230. 14998693 10.1016/j.survophthal.2003.12.007

[aos16776-bib-0037] van der Veen, I. , Heredero Berzal, A. , Koster, C. , Ten Asbroek, A. , Bergen, A.A. & Boon, C.J.F. (2024) The road towards gene therapy for X‐linked juvenile retinoschisis: a systematic review of preclinical gene therapy in cell‐based and rodent models of XLRS. International Journal of Molecular Sciences, 25, 1267. 38279267 10.3390/ijms25021267 PMC10816913

[aos16776-bib-0038] Verbakel, S.K. , van de Ven, J.P. , Le Blanc, L.M. , Groenewoud, J.M. , de Jong, E.K. , Klevering, B.J. et al. (2016) Carbonic anhydrase inhibitors for the treatment of cystic macular lesions in children with X‐linked juvenile retinoschisis. Investigative Ophthalmology & Visual Science, 57, 5143–5147. 27699410 10.1167/iovs.16-20078

[aos16776-bib-0039] Walia, S. , Fishman, G.A. , Molday, R.S. , Dyka, F.M. , Kumar, N.M. , Ehlinger, M.A. et al. (2009) Relation of response to treatment with dorzolamide in X‐linked retinoschisis to the mechanism of functional loss in retinoschisin. American Journal of Ophthalmology, 147(1), 111–115. 18834580 10.1016/j.ajo.2008.07.041 PMC2668603

[aos16776-bib-0040] Wang, N.K. , Liu, L. , Chen, H.M. , Tsai, S. , Chang, T.C. , Tsai, T.H. et al. (2015) Clinical presentations of X‐linked retinoschisis in Taiwanese patients confirmed with genetic sequencing. Molecular Vision, 21, 487–501. 25999676 PMC4415592

[aos16776-bib-0041] Weber, B.H. , Schrewe, H. , Molday, L.L. , Gehrig, A. , White, K.L. , Seeliger, M.W. et al. (2002) Inactivation of the murine X‐linked juvenile retinoschisis gene, Rs1h, suggests a role of retinoschisin in retinal cell layer organization and synaptic structure. Proceedings of the National Academy of Sciences of the United States of America, 99, 6222–6227. 11983912 10.1073/pnas.092528599 PMC122930

[aos16776-bib-0042] Wood, E.H. , Lertjirachai, I. , Ghiam, B.K. , Koulisis, N. , Moysidis, S.N. , Dirani, A. et al. (2019) The natural history of congenital X‐linked retinoschisis and conversion between phenotypes over time. Ophthalmol Retina, 3, 77–82. 30935660 10.1016/j.oret.2018.08.006

[aos16776-bib-0043] Xiao, Y. , Liu, X. , Tang, L. , Wang, X. , Coursey, T.G. , Guo, X. et al. (2016) X‐linked retinoschisis: phenotypic variability in a Chinese family. Scientific Reports, 6, 20118. 26823236 10.1038/srep20118 PMC4731765

[aos16776-bib-0044] Yang, H.S. , Lee, J.B. , Yoon, Y.H. & Lee, J.Y. (2014) Correlation between spectral‐domain OCT findings and visual acuity in X‐linked retinoschisis. Investigative Ophthalmology & Visual Science, 55, 3029–3036. 24713485 10.1167/iovs.14-13955

[aos16776-bib-0045] Zhao, C. , Zhang, Q. , Jin, H.Y. & Zhao, P.Q. (2018) Clinical observations of vitreoretinal surgery for four different phenotypes of X‐linked congenital retinoschisis. International Journal of Ophthalmology, 11, 986–990. 29977812 10.18240/ijo.2018.06.15 PMC6010392

