# Supplementary material for: A retrospective longitudinal study of 52 Finnish patients with X‐linked retinoschisis
Source: Acta Ophthalmol. 2024 Oct 22;103(2):196–204. doi: 10.1111/aos.16776 (PMC11810562; doi:10.1111/aos.16776)
Supplement: Supplementary file 1 — Table S1. [file AOS-103-196-s002.docx]

**Supplementary Table S1**. Distribution of different types of retinal characteristics in X-linked retinoschisis

| Macular schisis | Macular atrophy | Peripheral schisis | No of patients *  n=61 | No of eyes (%)  n=104 |
| --- | --- | --- | --- | --- |
| x | - | - | 33 | 59 (57%) |
| - | x | - | 4 | 5 (5%) |
| - | - | x | 0 | 0 (0%) |
| x | x | - | 7 | 12 (12%) |
| - | x | x | 1 | 1 (1%) |
| x | - | x | 9 | 14 (13%) |
| x | x | x | 4 | 7 (7%) |
| - | - | - | 3 | 6 (6%) |
| * at least one eye | | | | |
