# Supplementary material for: A retrospective longitudinal study of 52 Finnish patients with X‐linked retinoschisis
Source: Acta Ophthalmol. 2024 Oct 22;103(2):196–204. doi: 10.1111/aos.16776 (PMC11810562; doi:10.1111/aos.16776)
Supplement: Supplementary file 2 — Table S2. [file AOS-103-196-s005.docx]

**Supplementary Table S2.** Frequency of autorefraction (AR) and manual refraction (MR) in the patients with X-linked retinoschisis.

|  | AR baseline (n=85) | | AR last visit (n=53) | | MR baseline (n=86) | | MR last visit (n=63) | |
| --- | --- | --- | --- | --- | --- | --- | --- | --- |
|  | Frequency | Per cent | Frequency | Per cent | Frequency | Per cent | Frequency | Per cent |
| **Myopia**  ≤-0.5D | 8 | 9.5 | 2 | 4 | 9 | 10 | 11 | 17.5 |
| **Emmetropia** > −0.5D to ≤ +0.5D | 8 | 9.5 | 14 | 26 | 26 | 30 | 28 | 44.5 |
| **Hypermetropia** > +0.75 | 69 | 80 | 37 | 70 | 51 | 60 | 24 | 38 |
