# Supplementary material for: A retrospective longitudinal study of 52 Finnish patients with X‐linked retinoschisis
Source: Acta Ophthalmol. 2024 Oct 22;103(2):196–204. doi: 10.1111/aos.16776 (PMC11810562; doi:10.1111/aos.16776)
Supplement: Supplementary file 3 — Table S3. [file AOS-103-196-s001.docx]

| Supplementary Table S3. Correlation of refraction and optical coherence tomography of the macula in the patients with X-linked retinoschisis | | |
| --- | --- | --- |
| Refraction and OCT characteristics | ***Value*** | ***P-value*** |
| AR with CMT | *R*=0.133 | 0.64 |
| AR with OS length | *R*=0.366 | 0.20 |
| AR with EZ disruption | t(18)-1.745 | 0.09 |
| SR with CMT | *R*=0.137 | 0.58 |
| SR with OS length | *R*=-0.055 | 0.83 |
| SR with EZ disruption | t(19)=-0.431 | 0.67 |

OCT = optical coherence tomography, AR = autorefraction, SR = subjective refraction, CMT = central macular thickness, OS = photoreceptor outer segment, EZ = ellipsoid zone
