# Supplementary material for: A retrospective longitudinal study of 52 Finnish patients with X‐linked retinoschisis
Source: Acta Ophthalmol. 2024 Oct 22;103(2):196–204. doi: 10.1111/aos.16776 (PMC11810562; doi:10.1111/aos.16776)
Supplement: Supplementary file 4 — Table S4. [file AOS-103-196-s003.docx]

**Supplementary Table S4** Pathogenic variants, age at the time of diagnosis, initial and last visit, and number of visits during follow up years of 52 patients with X-linked retinoschisis

| Patient ID | Age at the time of diagnosis | | Pathogenic variant | Protein | Age at first visit | | Age at last visit | | No of visits |
| --- | --- | --- | --- | --- | --- | --- | --- | --- | --- |
| XLRS021 | | 1 | c.214>A | p.(Glu72Lys) | | 1 | 4 | 4 | |
| XLRS026 | | 1 | c.214>A | p.(Glu72Lys) | | 1 | 1 | 1 | |
| XLRS076 | | 1 | c.214>A | p.(Glu72Lys) | | 0 | 3 | 6 | |
| XLRS019 | | 2 | c.214>A | p.(Glu72Lys) | | 1 | 10 | 10 | |
| XLRS048 | | 2 | c.214>A | p.(Glu72Lys) | | 2 | 12 | 9 | |
| XLRS079 | | 3 | c.214>A | p.(Glu72Lys) | | 3 | 9 | 9 | |
| XLRS035 | | 4 | c.214>A | p.(Glu72Lys) | | 3 | 17 | 13 | |
| XLRS078 | | 5 | c.214>A | p.(Glu72Lys) | | 5 | 11 | 9 | |
| XLRS016 | | 6 | c.214>A | p.(Glu72Lys) | | 6 | 8 | 4 | |
| XLRS031 | | 6 | c.214>A | p.(Glu72Lys) | | NA | 11 | 7 | |
| XLRS072 | | 6 | c.214>A | p.(Glu72Lys) | | 6 | 19 | 10 | |
| XLRS005 | | 7 | c.214>A | p.(Glu72Lys) | | 6 | 18 | 24 | |
| XLRS010 | | 7 | c.214>A | p.(Glu72Lys) | | 7 | 8 | 2 | |
| XLRS064 | | 7 | c.214>A | p.(Glu72Lys) | | 7 | 13 | 5 | |
| XLRS001 | | 9 | c.214>A | p.(Glu72Lys) | | 7 | 15 | 11 | |
| XLRS015 | | 10 | c.214>A | p.(Glu72Lys) | | 9 | 13 | 3 | |
| XLRS017 | | 11 | c.214>A | p.(Glu72Lys) | | 39 | 57 | 3 | |
| XLRS022 | | 13 | c.214>A | p.(Glu72Lys) | | 27 | 31 | 4 | |
| XLRS075 | | 13 | c.214>A | p.(Glu72Lys) | | 35 | 62 | 7 | |
| XLRS077 | | 15 | c.214>A | p.(Glu72Lys) | | 7 | 20 | 10 | |
| XLRS024 | | 31 | c.214>A | p.(Glu72Lys) | | 26 | 32 | 2 | |
| XLRS053 | | 46 | c.214>A | p.(Glu72Lys) | | 46 | 58 | 5 | |
| XLRS034 | | 56 | c.214>A | p.(Glu72Lys) | | 9 | 57 | 24 | |
| XLRS080 | | NA | c.214>A | p.(Glu72Lys) | | 45 | 52 | 2 | |
| XLRS014 | | 2 | c.221G>T | p.(Gly74Val) | | 3 | 18 | 21 | |
| XLRS023 | | 3 | c.221G>T | p.(Gly74Val) | | 6 | 42 | 16 | |
| XLRS058 | | 3 | c.221G>T | p.(Gly74Val) | | 37 | 46 | 7 | |
| XLRS039 | | 4 | c.221G>T | p.(Gly74Val) | | 4 | 8 | 6 | |
| XLRS008 | | 5 | c.221G>T | p.(Gly74Val) | | 5 | 9 | 8 | |
| XLRS033 | | 6 | c.221G>T | p.(Gly74Val) | | 6 | 8 | 5 | |
| XLRS071 | | 6 | c.221G>T | p.(Gly74Val) | | 6 | 14 | 8 | |
| XLRS067 | | 7 | c.221G>T | p.(Gly74Val) | | 6 | 28 | 3 | |
| XLRS065 | | 7 | c.221G>T | p.(Gly74Val) | | 9 | 16 | 10 | |
| XLRS070 | | 13 | c.221G>T | p.(Gly74Val) | | 13 | 35 | 9 | |
| XLRS002 | | NA | c.221G>T | p.(Gly74Val) | | 70 | 72 | 5 | |
| XLRS011 | | 5 | c.325>C | p.(Gly109Arg) | | 5 | 16 | 13 | |
| XLRS036 | | 5 | c.325>C | p.(Gly109Arg) | | 4 | 17 | 18 | |
| XLRS009 | | 6 | c.325>C | p.(Gly109Arg) | | 6 | 12 | 8 | |
| XLRS012 | | 7 | c.325>C | p.(Gly109Arg) | | 7 | 15 | 6 | |
| XLRS013 | | 8 | c.325>C | p.(Gly109Arg) | | 17 | 63 | 27 | |
| XLRS007 | | 9 | c.554C>A | p.(Thr185Lys) | | 8 | 10 | 4 | |
| XLRS059 | | 10 | c.554C>A | p.(Thr185Lys) | | 10 | 13 | 5 | |
| XLRS029 | | 13 | c.554C>A | p.(Thr185Lys) | | 21 | 34 | 5 | |
| XLRS060 | | 8 | c.272G>T | p.(Gly91Val) | | 8 | 8 | 1 | |
| XLRS068 | | 11 | c.272G>T | p.(Gly91Val) | | 11 | 11 | 1 | |
| XLRS051 | | 2 | c.488G >A | p. (Trp163*) | | 1 | 12 | 20 | |
| XLRS028 | | 18 | c.488G >A | p. (Trp163Ter) | | 18 | 22 | 3 | |
| XLRS041 | | 7 | c.579dupC | p.(IleHisfs70) | | 4 | 30 | 24 | |
| XLRS006 | | 44 | c.579dupC | p.(lle194fs) | | 44 | 57 | 3 | |
| XLRS073 | | 8 | c.421C>T | p.(Arg141Cys) | | 8 | 15 | 6 | |
| XLRS042 | | 4 | c.331G>C | p.(ala 111 pro) | | 4 | 17 | 22 | |
| XLRS074 | | 57 | c.3G>A | p.(Met1?) | | 8 | 58 | 5 | |
