# Supplementary material for: A retrospective longitudinal study of 52 Finnish patients with X‐linked retinoschisis
Source: Acta Ophthalmol. 2024 Oct 22;103(2):196–204. doi: 10.1111/aos.16776 (PMC11810562; doi:10.1111/aos.16776)
Supplement: Supplementary file 5 — Table S5. [file AOS-103-196-s004.docx]

| Patient |  | Medication | BCVA,  logMAR | | Course of treatment |
| --- | --- | --- | --- | --- | --- |
|  |  |  | OD | OS | Week (w)/ Month (m)/ Years (y) |
| 1 | Baseline* Follow-up** | Oral CAI | 0.22 0.15 | 0.4 0.15 | 2 w |
| 2 | Baseline Follow-up | Oral CAI | - | 0.4  0.4 | 5 m |
| 3 | Baseline Follow-up | Oral CAI | - | 0.42  0.48 | 5 m |
| 4 | Baseline Follow-up | Oral CAI | 0.90 0.82 | 0.90 0.72 | 6 m |
| 5 | Baseline Follow-up | Oral CAI | 0.6  0.72 | 0.56 0.6 | 9 m 3w |
| 6 | Baseline Follow-up | Oral CAI | 0.32 0.50 | 0.32 0.50 | 1 y 4m |
| 7 | Baseline Follow-up | Topical CAI | 0.60 NA | 0.49 NA | continues |
| * Commencement of treatment  ** Termination of treatment   - No treatment | | | | | |

**Supplementary Table S5.** Visual acuity before and after treatment with carbomic anhydrase inhibitors in the patients with X-linked retinoschisis

CAI = carbonic anhydrase inhibitors, BCVA = Best-corrected visual acuity, logMAR = Logarithm of the Minimum Angle of Resolution OD = Right eye, OS = Left eye
